# Recent Advances in Treatment Options for Childhood Acute Lymphoblastic Leukemia

**DOI:** 10.3390/cancers14082021

**Published:** 2022-04-16

**Authors:** Marta Malczewska, Kamil Kośmider, Kinga Bednarz, Katarzyna Ostapińska, Monika Lejman, Joanna Zawitkowska

**Affiliations:** 1 Department of Pediatric Hematology, Oncology, and Transplantology, Medical University of Lublin, Gębali 6, 20-093 Lublin, Poland; martmalczewska@gmail.com; 2 Student Scientific Society, Laboratory of Genetic Diagnostics, Medical University of Lublin, Gębali 6, 20-093 Lublin, Poland; kamilkosmider96@gmail.com (K.K.); kbedna.98@gmail.com (K.B.); katarzyna1ostapinska@gmail.com (K.O.); 3 Laboratory of Genetic Diagnostics, Medical University of Lublin, Gębali 6, 20-093 Lublin, Poland; monika.lejman@umlub.pl

**Keywords:** acute lymphoblastic leukemia, pediatric, conventional therapy, chemotherapy, radiotherapy, immunotherapy, targeted therapy, CAR-T

## Abstract

**Simple Summary:**

Acute lymphoblastic leukemia is the most common blood cancer in pediatric patients. Despite the enormous progress in ALL treatment, which is reflected by a high 5-year overall survival rate that reaches up to 96% in the most recent studies, there are still patients that cannot be saved. Treatment of ALL is based on conventional methods, including chemotherapy and radiotherapy. These methods carry with them the risk of very high toxicities. Severe complications related to conventional therapies decrease their effectiveness and can sometimes lead to death. Therefore, currently, numerous studies are being carried out on novel forms of treatment. In this work, classical methods of treatment have been summarized. Furthermore, novel treatment methods and the possibility of combining them with chemotherapy have been incorporated into the present work. Targeted treatment, CAR-T-cell therapy, and immunotherapy for ALL have been described. Treatment options for the relapse/chemoresistance ALL have been presented.

**Abstract:**

Acute lymphoblastic leukemia is the most common blood cancer in pediatric patients. There has been enormous progress in ALL treatment in recent years, which is reflected by the increase in the 5-year OS from 57% in the 1970s to up to 96% in the most recent studies. ALL treatment is based primarily on conventional methods, which include chemotherapy and radiotherapy. Their main weakness is severe toxicity, which prompts dose reduction, decreases the effectiveness of the treatment, and, in some cases, can lead to death. Currently, numerous modifications in treatment regimens are applied in order to limit toxicities emerging from conventional approaches and improve outcomes. Hematological treatment of pediatric patients is reaching for more novel treatment options, such as targeted treatment, CAR-T-cells therapy, and immunotherapy. These methods are currently used in conjunction with chemotherapy. Nevertheless, the swift progress in their development and increasing efficacity can lead to applying those novel therapies as standalone therapeutic options for pediatric ALL.

## 1. Introduction

Acute lymphoblastic leukemia is the most commonly diagnosed type of cancer in pediatric patients. It accounts for approximately 26% of malignances in children [1,2]. In the United States alone, there are about 6000 newly discovered ALL cases each year, the vast majority of which are diagnosed in males than in females (3:1 ratio) [3,4].

Overall survival (OS) and event-free survival (EFS) are the main indicators in the treatment of ALL. The enormous progress of ALL treatment is reflected by the increase in the 5-year OS from 57% in the 1970s to up to 96% in the most recent studies. Furthermore, currently the 5-year EFS reaches 92% [1,5,6,7,8,9,10,11,12].

According to the 2017 revision to the World Health Organization (WHO) classification of myeloid neoplasms and acute leukemia, based on the immunophenotype assessment, ALL cases can be classified as B-cell precursor ALL (B-ALL), which occurs in 85% of diagnosed patients and T-cell ALL (T-ALL), which accounts for the remaining 15% of cases [5,13,14].

**Figure 1:**
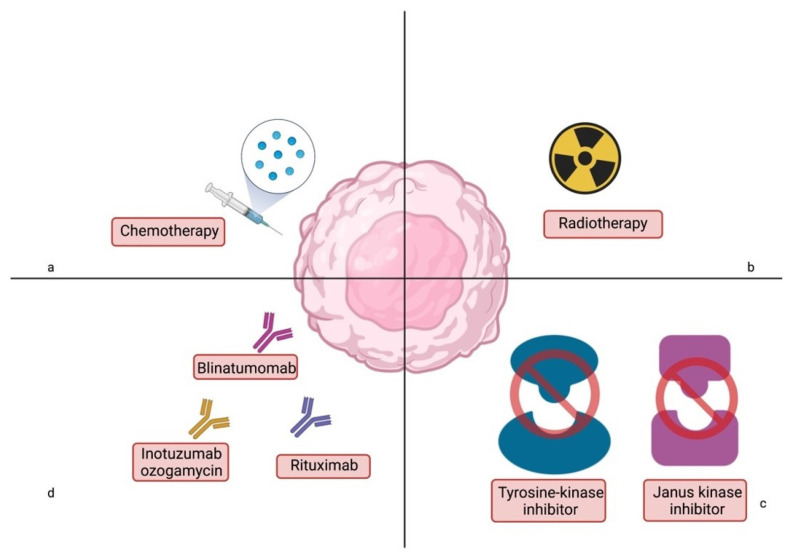
Current therapeutic options for acute lymphoblastic leukemia in children. Starting with conventional therapies: (**a**)—chemotherapy and (**b**)—radiotherapy; through targeted treatment via kinase inhibitors—(**c**); ending with the latest treatment methods—(**d**). Image created with biorender.com (accessed on 14 March 2022).

Currently, treatment with conventional methods has reached its limit mostly because of the tremendous toxicities that are seen in children during treatment. Modifications of the treatment regimens are used to reduce multiple toxicities. The introduction of safer therapies has become a priority in the management of hematological pediatric patients. Targeted therapy or immunological methods such as chimeric antigen receptor T (CAR-T) cells or bispecific T-cell engager (BiTE) are the perspectives for the not-too-distant future. These novel agents are a significant step in pursuing the goal of the 100% OS in childhood ALL [15]. When comparing cure rates for ALL in adults and children, only 30–40% of adults achieve remission. In adult patients, new immunological approaches are also being used, accompanying standard steroids and chemotherapy. In addition, due to the very high genetic heterogeneity, drugs that block specific metabolic pathways (such as TKIs, venetoclax, navitoclax) are being added. Intensive research is being conducted, especially on the possibility of combining treatment options. Significant improvements in survival rates (in both groups: over and under 60 years of age) are shown in studies where chemotherapy is combined with antibodies blinatumomab or inotuzumab ozogamicin [16]. Given the ongoing swift progress in the field of ALL therapy, advances in pediatric ALL treatment ranging from conventional therapy to immunotherapeutic agents have been presented in this review. The current therapeutic options for ALL are shown in Figure 1.

## 2. Conventional Therapy of ALL

As treatment of leukemia has improved over the past several decades, research has focused on looking for prognostic factors that influence a patient’s final response to treatment. Prognostic factors determine the assignment to a given risk group and thus the selection of the appropriate intensification of therapy. In developed countries, different protocols are used to treat ALL, and each of them distinguishes risk groups and prognostic factors: minimal residual disease (MRD), response to steroids therapy, immunophenotype, genetic factors, age of patient, white blood counts (WBC) at diagnosis used to qualify patients [17]. One of the most important prognostic factors in patients with ALL remains MRD status which is defined as persistent malignant cells resistant to the chemotherapy that is used. Detection of 1 neoplastic cell per 10,000 healthy cells (0.01%) is sufficient for a positive MRD result. Minimal residual disease informs about the effectiveness of the applied treatment and the patient’s chemosensitivity, which allows for determining the residual burden of the disease and the risk of relapse [18,19]. Detection of persistent leukemic cells is based on two methods: multiparametric flow cytometry (FC-MRD) and molecular methods based on polymerase chain reaction (PCR-MRD) [20]. FC-MRD and PCR-MRD status are determined at assigned time points (TP) during treatment. Multiparametric FC, based on the detection of leukemia-specific immunophenotypes using different combinations of fluorochrome-labeled monoclonal antibodies, is the fastest and most sensitive of approximately 10^−4^ methods [21]. Flow cytometry is applicable in about 90–98% of patients with ALL, but its sensitivity is lower as compared to methods based on real-time quantitative polymerase chain reaction (RQ-PCR) that is 10^−4^–10^−6^. The analysis of immunoglobulin (Ig) and the T-cell receptor (TCR) gene rearrangements are used as MRD markers in this technique (Ig/TCR-RQ-PCR) [22]. Recently, the Euro Clonality-NGS Working Group developed a method for next-generation sequencing (NGS)-based IG clonality analysis. The study by van den Brand et al. showed that NGS-based IG clonality analysis is a high interlaboratory concordance (99%) and high concordance with conventional clonality analysis (98%) for the molecular conclusion [23]. Another prognostic factor is the response to steroid therapy, which can be measured by testing the presence of peripheral blasts. It is measured after one week of steroid pro-phase. The group with the better results and, therefore, a greater chance of survival includes those who have a peripheral blast count less than 1000/mm^3^ (good response to steroids). Patients with a poor response to steroids with peripheral blasts ≥ 1000/mm^3^ are assigned a higher risk. Patients between 1 and 10 years old also have a significantly better overall prognosis [24]. High WBC count at diagnosis is also an adverse factor, as patients with WBC count ≥50,000/mm^3^ show worse response to treatment. In contrast to patients with pre-B-ALL, age and WBC count play less of a role in determining prognosis in patients with T-cell ALL (T-ALL) [25]. Genetic abnormalities in a cancer cell also affect a patient’s prognosis. Hyperdiploid karyotype or *ETV6*::*RUNX1* rearrangements are associated with a good prognosis. Hypodiploid karyotype, *BCR*::*ABL1*, *KMT2A* rearrangement, *TCF3*::*HLF* fusion gene, or Ph-ALL-like subtype are connected with the poor prognosis. Intermediate prognosis is associated with *TCF3*::*PBX1* fusion or newly identified *ETV6*::*RUNX1*-like rearrangement. Some of them are suitable candidates for molecular targeted therapy.

First-line treatment of pediatric ALL is conducted in several phases such as induction, consolidation, intensification (reinduction, delayed intensification in some protocols), and remission maintenance therapy. Induction of remission is based on steroid therapy accompanied by administration of cytostatic. Prednisone and dexamethasone were among the first drugs used in the treatment of ALL and remain an essential part of therapy. Consolidation of remission and maintenance therapy in most protocols is based on systemic and intrathecal cytostatic administration. Steroids have the ability to bind to the glucocorticoid receptor, resulting in inhibition of cytokine production, altered oncogene expression, cell cycle inhibition, and eventually cell apoptosis [26]. The general principle of cytostatic agents is to disrupt the cell cycle and cause cell death or inhibit cell growth and division. The most commonly used are methotrexate, daunorubicin, doxorubicin, vincristine, cytarabine, cyclophosphamide, thioguanine, and 6-mercaptopurine. The entire therapy usually takes about 2–3 years. Over the years, many research groups have undertaken studies to determine the effectiveness of specific protocols and their modifications in treating pediatric ALL patients. A large study, AIEOP-BFM ALL 2000, involving 127 centers, took place between July 2000 and July 2006. The study enrolled 4839 children and adolescent patients from the ages of 1 to 17 years and diagnosed with ALL. The 5-year EFS was 83.9%, and OS was 90.5%. Multiple toxicities, including life-threatening events, were registered in this study. The most frequent one was the development of bacterial infection (43%) during the induction phase. As many as 43% of these infections turned out to be fatal [5,27].

The UKALL 2003 trial was going on between October 2003 and June 2011. This study enrolled 3207 children and young adults (to 25 years old) with ALL. The 5-year EFS was 87%, and the 5-year OS was 91.5%. In this study, 1101 serious adverse events were reported, and 16% of them were serious infections. Bacterial infections were the most common, followed by fungal infections. During the induction phase, 17 deaths were reported, mainly due to infection [28]. The study of Children Oncology Group (COG) AALL0331 included 5377 patients between the age of 0 and 10 and took place between April 2005 and May 2010. The 6-year EFS was 88.96%, and OS rates were 95.54%. The rate of grade 3 and 4 neutropenia was 62%, while neutropenic fever was 29%. Infections accounted for 23% of reported adverse events [29]. From August 2002 to October 2009, a total of 150 patients with B-cell ALL aged from 15 to 24 years were enrolled in the Japan Association of Childhood Leukemia Study (JACL’s) ALL202-U. The EFS and OS at 5 years for all patients were 67% and 73%. About 100% of patients developed grade 4 neutropenia during induction. A total of 46.5% experienced neutropenic fever, and nearly 5% developed sepsis as a result of infection [30]. Currently, there are several protocols for the treatment of pediatric ALL, and more than a dozen studies are ongoing. Table 1 shows the differences between the selected protocols used in developed countries.

In addition to chemotherapy, radiation is used to treat acute lymphoblastic leukemia in select groups of patients. Formerly, craniospinal irradiation was a crucial departure point in the treatment of leukemia. Currently, eligibility for radiation therapy depends on a specific central nervous system (CNS) status at diagnosis. The criteria for CNS involvement are, subsequently: status 1—absence of CNS involvement; status 2—pleocytosis ≤ 5/µL with clearly identified blasts on cytospin of cerebro-spinal fluid (CSF) contaminated with blood; status 3—non-traumatic lumbar puncture with pleocytosis >5/µL or damage to the brain/meninges seen in imaging studies or the presence of neurological symptoms [36]. Due to CNS involvement in ALL predisposition to poor treatment outcomes, cranio-spinal irradiation is used to control CNS recurrence [37]. CNS irradiation is usually used in high-risk ALL patients with CNS status 3 [38]. Due to the high toxicity, research has begun on reducing the radiation dose, the time at which the patient receives radiotherapy, or whether radiotherapy should be included in treatment at all. The concept behind the ALL-BFM trial protocols conducted between 1981 and 2000 was to minimize therapy to the point where disease control could be achieved without overwhelming side effects. Since 1981, irradiation doses have been reduced. In the ALL-BFM 83 study, prophylactic cranial radiotherapy (pCRT) was used at doses of 12 Gy and 18 Gy. The results indicated the efficacy of the lower irradiation dose. In ALL-BFM 95, pCRT was no longer used in patients with non-T-ALL, except for HR patients [39]. In protocol ALL IC BFM2002, only patients with T-ALL and HR at 1 year of age received cranial prophylaxis radiotherapy (12 Gy) [36]. The aforementioned trial focused on infant ALL, INTERFANT-99 did not use the cranial irradiation on its subjects due to proven neurocognitive repercussions of high severity. In this case, young age is considered a crucial factor predisposing to the adverse effects [40,41,42]. The results from the AALL02P2 study conducted by the Children Oncology Group (COG) were published in 2021. The aim of the study was to perform intensified systemic treatment using drugs that penetrate highly into the CNS (dexamethasone, high doses of cytarabine, and MTX), which allowed delaying CRT by 12 months and reducing the irradiation dose to 12 Gy. The outcome of the study was for patients with B-ALL and isolated central nervous system relapse (*n* = 112). In previous studies conducted by COG-POG 9061 and POG 9412, patients received radiotherapy at doses of 24 and 18 Gy and delayed radiotherapy for 6 to 12 months. The 3-year EFS and OS for patients on COG AALL02P2 (*n* = 118) were 64.3% ± 4.5% and 79.6% ± 3.8%. Compared to late CNS-R B-ALL patients (*n* = 50) on POG 9412 (previous study conducted by the COG), the 3-year EFS for COG AALL02P2 was significantly inferior (64.3% ± 4.5% compared to 79.9% ± 5.8%; *p* = 0.03). The 3-year cumulative recurrence rate was 26.7%, with a higher incidence in HR patients. These studies demonstrate that CNS involvement is still a challenge in the treatment of ALL, and radiotherapy significantly improves outcomes in these patients. However, dose reduction and delayed radiotherapy should be continuously pursued to reduce toxicity [43]. For patients with high-risk features in first complete remission (CR1), refractory, or relapsed disease, the highly effective treatment option remains allogeneic hematopoietic stem cell transplantation (HSCT). This group of selected patients includes children who have genetic anomalies that are associated with poor prognosis, for example: t(9;22) *BCR*::*ABL1*, t(4;11) *KMT2A*::*AFF1*, intra-chromosomal amplification of chromosome 21 [44]. Most children, prior to allogeneic hematopoietic stem cell transplantation, receive conditioning that includes total body irradiation (TBI). Since TBI is associated with huge complications that affect the adult life of patients (i.e., growth impairment, hypothyroidism and delayed puberty, infertility), studies have been initiated on the necessity of TBI. So far, it has not been proven that TBI can be successfully replaced by chemotherapy exclusively. The most recent report by Styczynski et al. on the issue of using TBI or chemotherapy before transplantation was published in 2020. A total of 139 cases were analyzed: 55 patients underwent conditioning with TBI, and the remaining 84 patients were initially treated with chemotherapy alone. The 2-year OS was achieved by 84% after TBI, compared with only 60.5% after chemotherapy conditioning [45]. The results were confirmed by the outcome of a study published in 2021 involving 3045 patients. Patients were divided into two groups: patients in complete remission 1 (CR1) and in complete remission 2 (CR2). Out of group 1 (*n* = 1498), 1285 patients received TBI, and 213 patients received chemotherapy before HSCT. Among group 2 (*n* = 1556), 1345 patients received TBI, and 211 patients received chemotherapy before HSCT. The EFS rate in both groups was higher in patients who received TBI than in patients who received chemotherapy; in the CR1 group, 63.8% vs. 61.4%, and in the CR2 group, 53.7% vs. 29.4% [46]. The phase III, randomized, controlled, open-label, international, multicenter For Omitting Radiation Under Majority age (FORUM) study recruited 417 pediatric high-risk ALL patients in CR prior to the HSCT. Patients were randomly assigned into two groups. The first group received busulfan-based chemoconditioning, whereas the second group underwent TBI. TBI was proven to be more beneficial, as the 2-year OS of patients who were assigned to the TBI group was significantly higher than the 2-year OS of the remaining participants (91% vs. 75%, *p* < 0.0001). Similarly, 2-year EFS was also higher in the TBI group than in the patients who were given conditioning chemotherapy (86% vs. 58%, *p* < 0.0001). TBI conditioning was also associated with a lower risk of relapse, as the 2-year cumulative incidence of relapse (CIR) rate was lower in this group (12% vs. 33%, *p* < 0.0001) [47].

The effectiveness of HSCT has been demonstrated in many studies. For example, in 2018, a study report of 119 patients aged 1 to 18 years who received HSCT at AIEOP transplant centers was published. The aim of the study was to quantify MRD in patients immediately after transplantation as well as in the third trimester after bone marrow transplant. After HSCT, 61 patients from the study group achieved disease-free status, which allowed for the estimation of EFS and the 10-year OS at the level of 50% and 54%, respectively. Forty-eight patients relapsed after transplantation, with a median duration of 7 months after transplantation [48]. In light of the emergence and increasing use of new therapeutic approaches (i.e., blinatumomab, CART-cell) in the context of post-HSCT or TBI-alone complications, the role of HSCT in ALL treatment should be re-examined.

## 3. Targeted Therapies Used in ALL

The genetic characterization of ALL has revolutionized clinical treatment approaches. Currently, childhood ALL can be divided into more than 30 genetic subgroups that are associated with individual patient prognosis. Several groups in which the presence of a mutation or chromosome translocation leads to activation of particular metabolic pathways can be distinguished. This is usually associated with a poor diagnosis, as these pathways are commonly associated with increased cell survival. Nevertheless, over the years, methods of targeting those overactivated cascades in ALL have been developed. Mitigating consequences of overexpressed pathways significantly improve prognosis in previously HR groups. Furthermore, agents targeting signaling cascades contribute to decreased treatment toxicity.

### 3.1. Tyrosine Kinases Inhibitors

The Philadelphia chromosome occurs in approximately 3–5% of patients with ALL. It is associated with a worse prognosis; before the introduction of targeted therapy, it was only 30% [49]. ALL Ph+ patients carry a translocation between chromosomes 9 and 22, which leads to the fusion of the *BCR* and *ABL* genes. This is followed by activation of the tyrosine kinases ABL1 and ABL2, which transduce signals through multiple pathways such as: the JAK/STAT pathway, the mTOR pathway, the MAPK/ERK pathway, the TRIAL-included pathway, and the differentiation pathway mediated by CEBP [50]. Tyrosine kinases mediate signal transduction by catalyzing the transfer of adenosine triphosphate (ATP) to the substrate protein. Oncogenic tyrosine kinase can be inhibited with targeted compounds, tyrosine kinase inhibitors (TKI). TKIs can be classified as type I or type II inhibitors, depending on whether they recognize an active or inactive kinase conformation. Type I inhibitors directly compete for binding to ATP, while type II inhibitors are competitive with ATP. Tyrosine kinases inhibitors can also be divided into generations, depending on when they were introduced for treatment [51]. The first trial using the I generation TKI—imatinib took place in 2004. The drug was added to the standard protocol after the consolidation phase. In a subsequent study in 2010, imatinib was administered from day 15 of chemotherapy induction [52,53]. Along with advances in the treatment of this type of leukemia, there has arisen a group of patients who develop intolerance or resistance to imatinib. Therefore, next-generation TKIs have been introduced for treatment. Second-generation TKi used to treat acute lymphoblastic leukemia includes Dasatinib and Nilotinib. Dasatinib has the ability to cross the blood-brain barrier and has greater activity against mutations responsible for imatinib resistance. The Chinese Children’s Cancer Group (CCCG) in 2014 started a clinical trial with 189 patients. They were divided into two groups: one of them (*n* = 97) was receiving imatinib, and the second one (*n* = 92) dasatinib (II generation TKI). The group of patients who were administered dasatinib had significantly higher rates: the 4-year event-free survival (71.0% vs. 48.9%) and overall survival (88.4% vs. 69.2%) [52]. Despite the introduction of second-generation TKI, there were still patients who developed resistance. In most of them, point mutations in the *BCR*::*ABL* fusion gene were found [54]. Due to this phenomenon, it was necessary to develop a drug that has the ability to overcome mutations engineered by the tumor cell, primarily the T315I mutation, also knowns as the “gatekeeper mutation”. It causes a hydrogen bonding break, which is critical for drug binding and responsible for resistance to first- and second-generation TKIs. In a 2015 study involving 37 patients who were treated with ponatinib; OS was 80%. Ponatinib is a third-generation TKI that has become a great hope for Ph+ patients who have developed intolerance or resistance to other generations of TKIs [55]. Because this group of patients is constantly increasing, new generations of drugs are being introduced for treatment.

### 3.2. Ruxolitinib

The interleukin-7 (IL-7)/Janus kinase (JAK)/signal transducer and activator of transcription (STAT) signaling pathway is involved in both T and B-cells’ development [56,57,58,59]. Furthermore, STAT proteins play a vital role in cells’ survival, as they increase the synthesis of antiapoptotic Bcl-2 protein [60,61]. The IL-7/JAK/STAT pathway has been shown to be activated in T-ALL in both patient-derived leukemic cells and T-ALL cell lines, which was associated with increased cancer cell survival [62,63,64,65]. Therefore, JAK1/2 inhibitor ruxolitinib was tested during the preclinical studies. In a study by Maude et al., patient-derived xenografts models of early T-cell precursor (ETP) ALL have been established in NOD/SCID mice. Ruxolitinib significantly (*p* < 0.01) reduced blast count in 5 of 6 xenograft models. Importantly, the efficiency of ruxolitinib was independent of the presence of JAK/STAT activating mutations; thus, it was suggested that this drug can be used in a larger group of patients than it was expected [66]. In a study by Böhm and colleagues, the results of ruxolitinib treatment in Ph-like ALL preclinical models have been evaluated. Overactivation of JAK/STAT occurs in most of the Ph-like ALL cases. Of particular importance are rearrangements that overexpress the cytokine receptor-like factor 2 (CRLF2) gene, resulting in inappropriate activation of JAK signaling. The combination treatment with vincristine, dexamethasone, L-asparaginase, and ruxolitinib of mice engrafted with CRLF2-rearranged Ph-like ALL patient-derived xenografts decreased leukemic infiltration of various organs and prolonged time to remission, as compared to chemotherapy or ruxolitinib alone [67]. Ruxolitinib is currently involved in a number of studies, such as Total Therapy Study XVII, which include ruxolitinib for the induction phase in the patients with activation of the JAK/STAT pathway (NCT03117751). Currently, ruxolitinib in cotreatment with consolidation chemotherapy is also being tested in the treatment of children with HR CRLF2-rearranged and/or JAK pathway-mutant Ph-like pre-B-ALL (NCT02723994). Moreover, the phase II study of ruxolitinib in combination with chemotherapy for relapsed/remitting Ph-like ALL has been completed, and the results are yet to be posted (NCT02420717).

## 4. Immunotherapies in Pediatric ALL

### 4.1. Antibodies

Novel targeted therapies are currently used in order to support conventional cancer therapy. The innovative approaches are implemented to reduce the toxicity burden and improve outcomes. Monoclonal antibodies represent a group of new, targeted drugs aiming at specific antigens presented by the neoplastic cells. Those drugs induce cytotoxicity by stimulating the immune system against cancer cells. Nowadays, the most common targets in pediatric ALL patients are: CD19, CD20, and CD22. Antibody-based drugs aiming for these antigens cover a range of monoclonal antibody-based treatments, as well as treatment approaches based on antibody-drug conjugates.

#### 4.1.1. Bispecific Anty-CD19 and Anty-CD3 Antibody

For now, the only registered FDA monoclonal antibody is blinatumomab, a bispecific T-cell engager (BiTE) that comprises two antibodies directed against CD19 and CD3. CD19 is expressed on all precursor B-cells, whereas CD3 is a mediator of T-cell-dependent signaling, and this combination enables the strong engagement of T-cells and thus the proliferation of activated T-cells and tumor cell lysis [68].

Blinatumomab-based therapy was applied in different subtypes of relapsing/remitting childhood ALL in March 2021. The results of two randomized blinatumomab trials involving B-ALL-pediatric patients have been published. Locatelli et al. reported a trial enrolling 108 participants between 28 days and 18 years old. Patients received induction chemotherapy and two cycles of consolidation chemotherapy, after which they were randomized. The patients had morphologic CR with M1(<5% blasts) or M2 (between ≥5% and <25% blasts) bone marrow at the time of randomization. Blinatumomab was compared to conventional chemotherapy. Patients were divided into equal groups that received either one cycle of blinatumomab or three cycles of consolidation chemotherapy. Beneficial effects of blinatumomab were observed, with a favorable safety profile (adverse events rate 31% vs. 57%), more often MRD remission (90% vs. 54%), and lower mortality (14.8% vs. 29.6%). The OS hazard ratio after a median follow-up time of 19.5 months was 0.43 (95% CI, 0.18–1.01) in the blinatumomab group, whereas in the patients who received chemotherapy, the OS hazard ratio was 0.35 (95% CI, 0.12–1.01). Furthermore, more blinatumomab-treated patients were eligible to proceed with HSCT (88.9% vs. 70.4%) [69]. A trial conducted by Brown et al. investigated the post-reinduction consolidation therapy prior to the HSCT, including either blinatumomab or chemotherapy in 219 patients aged 1–30 years old with high- and intermediate-risk first relapse of B-ALL. Each group received a 4-week scheme of reinduction chemotherapy followed by either two cycles of blinatumomab or multiagent chemotherapy, each prior to HSCT. Advantages of blinatumomab treatment were reflected by the improved 2-year OS rate (71.3% vs. 58.4%) and a higher rate of 2-year disease-free survival (54.4% vs. 39.0%). Furthermore, blinatumomab was characterized by a beneficial safety profile [70]. Therefore, blinatumomab was identified as a beneficial agent in treating relapsed B-ALL. Improved outcomes and safety profile in comparison to chemotherapy indicate that this drug may be soon introduced into the treatment protocols. Currently, a significant number of studies regarding the effects of blinatumomab administration in B-ALL is being conducted (e.g., NCT04994717, NCT04521231, NCT02003222, NCT04530565).

#### 4.1.2. Antibody-Drug Conjugates Targeting CD19

SAR3419 (coltuximab ravtansine), an antibody-drug conjugate of maytansinoid DM4 and anti-CD19 antibody, has proven its efficacy preclinically in B-ALL and infant mixed-lineage leukemia (MLL) immune-deficient (NOD/SCID) mice xenografts. Coltuximab ravtansine has been demonstrated to significantly delay the disease progression in all xenografts and induce an objective response in six out of seven xenografts. SAR3419 also prevented relapse in hematolymphoid tissue and other organs (with the exception of the CNS system). Therefore, it was suggested that SAR3419 may improve the outcomes in pediatric CD19+ B-ALL [71].

Denintuzumab mafodotin (SGN-CD19A) is also an interesting antibody-drug conjugate targeting CD19. It is comprised of a humanized monoclonal anti-CD19 antibody with monomethyl auristatin F (an anti-mitotic agent). Jones et al. conducted a study in which B-lineage patient-derived xenografts were established in NOD/SCID mice. The animals were given denintuzumab mafodotin orally for 3 weeks. In all of the animals, CR was achieved, proving that denintuzumab mafodotin may be a suitable agent in treating B-lineage ALL [72]. Denintuzumab mafodotin has demonstrated antileukemic activity during phase I clinical study by Fathi et al. During the study, 9 of 55 adult B-ALL patients achieved CR, 3 patients achieved CR without platelet recovery and an additional 2 CR with incomplete blood recovery. The treatment was well tolerated, and obtained results indicate that it may prove useful in B-ALL treatment [73].

### 4.2. Anti-CD20 Antibodies

CD20 is expressed on the surface of all mature B-cells. It is encoded by the MS4A1 gene, and it has an essential role in the development and maturation of B-cells, along with providing the optimal function of B-cells. This particular B-specific antigen currently constitutes a second-line target for B-ALL therapies; approximately 25% of all patients qualify for anti-CD20 immunotherapy [74,75].

#### Rituximab

Rituximab is a chimeric monoclonal antibody comprising the human and murine compounds directed against CD20. The mechanism of action characteristic of rituximab is the depletion of leukemic B-lineage cells via immune-mediated cytotoxicity followed by the induction of apoptosis. Despite the targeted character of rituximab therapy, it is associated with certain risks and secondary adverse events, as rituximab suppresses the physiological B-cell count and reduces the number of circulating antibodies. To date, the efficiency of rituximab has not been comprehensively assessed in pediatric cohorts. Fortunately, the beneficial effect of rituximab in combination with chemotherapy has been proven in young adults with B-ALL [76]. Preclinical data obtained on rituximab and its contenders suggest that another anti-CD20 monoclonal antibody, obinutuzumab, may be superior to rituximab, as it was characterized by the relatively better performance during the preclinical studies [77].

### 4.3. Anti-CD22 Antibodies

As CD22 antigens are presented by around 80% to 90% of B-ALL cells, they have become another target for immunotherapy. Antibodies targeting this antigen are epratuzumab and moxetumomab pasudotox. A phase II clinical trial was conducted to evaluate the efficacy of moxetumomab pasudotox in children with B-ALL. Unfortunately, the study was terminated ahead of schedule as the first 32 subjects enrolled in the study did not achieve the required efficacy (NCT02227108). Epratuzumab has been tested for its effects in combination with reinduction chemotherapy in 114 patients from 2 to 30 years old. Patients received three reinduction blocks according to the AALL01P2 regimen. The participants were divided into two groups who were given epratuzumab with the first block of reinduction chemotherapy: the first group (B1) received one dose of epratuzumab per week (*n* = 54), while the second (B2) received epratuzumab twice a week (*n* = 60). The OS and the 2-year survival rate were evaluated for both groups. For group B1 the OS ratio was 34.2 ± 6.9%, while for group B2 it was 49.3 ± 8.1%. For groups B1 and B2, the 2-year EFS was 25.9 ± 6.4% and 39.9 ± 8.0%, respectively. There was no significant improvement in achieving second CR as compared to the historical control group (COG AALL01P2), in which chemotherapy alone was used. Nevertheless, in the early relapse patients (18–36 months after the diagnosis), the addition of epratuzumab to the classical chemotherapy improved MRD-negative CR in the B2 group (44% vs. 25% in the historical control group). Unfortunately, due to the small sample size, this beneficial effect was statistically insignificant (*p* = 0.1215) [78]. Currently, moxetumomab pasudotox is being tested during the phase III study with the participation of children with relapsed ALL (NCT01802814).

Another agent targeting the CD22 antigen is inotuzumab ozogamicin. It is a humanized antibody-drug conjugate consisting of calicheamicin cytotoxin and kappa immunoglobulin G4 [79,80]. In 2018, the results of a retrospective multinational study conducted on a group of 51 pediatric patients (≤21 years old) with relapsed/remitting B-ALL were published. Patients received at least one dose of inotuzumab ozogamicin during the study. The 1-year OS ratio was 36.3 ± 9.3%, and the EFS was 23.4 ± 7.5%. Overall, 28 patients achieved CR. Most of the patients (86%) achieved CR after the first cycle of treatment. Of the patients with CR, 71% were MRD-negative (*n* = 20) [81]. A similar CR rate was obtained in the other studies involving inotuzumab ozogamicin for pediatric B-ALL, conducted by the French and Spanish groups [82,83].

### 4.4. Chimeric Antigen Receptor T-Cells

**Figure 2:**
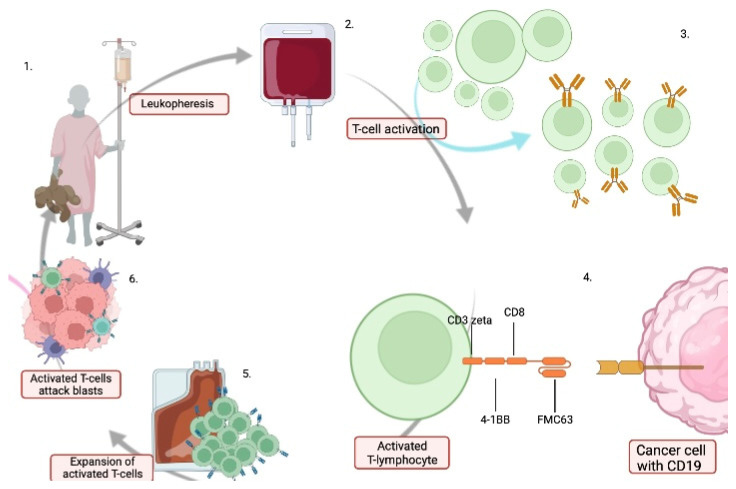
Steps of the CART-cell therapy. 1—Peripheral blood mononuclear cells are collected from the patient by plasmapheresis. 2—The leukapheresis material is cryopreserved and transported to the laboratory. 3—T-cells are activated with beads that are coated with anti-CD3/CD28 antibodies. They are then transduced with a self-activating vector containing an anti-CD19 CAR transgene. 4—There is a CD19 antigen on the tumor cell. Opposite the tumor cell is the CAR tisagenlecleucel. It consists of a single-chain CD19-specific mouse antibody fragment (FMC63), a CD8-a hinge, and a transmembrane region linked to domains: CD3-zeta (signaling) and 4-1BB (costimulatory) domain. The 4-1BB domain serves as a costimulatory signal for cell activation. 5—Cell multiplication is carried out until sufficient numbers are reached. 6—The finished product, which has the ability to attack blastic cells, is administered to the patient. Image created with biorender.com (accessed on 14 March 2022).

Chimeric antigen receptor T (CAR-T) cells are patient-derived, genetically engineered T-cells, which, after transformation, are applied back to the patient. During modification, lymphocytes gain extracellular domains, which bind to the antigens on tumor cells, triggering a T-cell response [84]. Figure 2 shows the process of applying CAR-T therapy.

The most common CAR-T target is CD-19, which is expressed on almost all lymphoblasts [84]. In 2017, the FDA (U.S. Food and Drug Administration) approved the anti-CD19 CAR-T-based drug tisagenlecleucel for the treatment of B-ALL in patients <26 years of age [85].

In 2015, the results of a phase I trial, which involved 21 patients aged 1 to 30 years, including 20 patients with relapsing/recurring B-ALL, were published. Prior to a single CAR-T infusion, all patients received cyclophosphamide and fludarabine. The dose-escalation part of the study has been designed according to the standard 3 + 3 design. Patients received either 1 × 10 × 6 CAR-T-cells per kg or 3 × 10 × 6 CAR-T-cells per kg. The entire dose of produced lymphocytes was administered in case a sufficient number of CAR-T-cells had not been generated. Nevertheless, such patients have not been involved in dose escalation; however, they were assessed for toxicity and for other parts of the study. The maximum tolerated dose of 1 × 10 × 6 cells per kg was established, given the dose-limiting toxicities in two out of four patients who received 3 × 10 × 6 CAR-T-cells/kg. Such toxicities have not been observed in the patients who received lower doses. Therefore, the dose of 1 × 10 × 6 CAR-T-cells per kg was used in the expansion of the study (patients 11–21). Overall, 28.6% (6/21) of patients experienced grade 3/4 cytokine release syndrome (CRS), which is a serious complication related to CAR-T therapy, which can potentially lead to multiorgan failure and death. A total of 14 (70%) patients with relapsing/recurring B-ALL achieved a complete response (CR), out of which 12 achieved MRD-negative status. Further, 10 of them were qualified for allo-HSCT transplants. All of these patients remain disease-free. In contrast, the remaining 2 of the patients with MRD-negative status who did not qualify for allo-HSCT relapsed with CD19-negative leukemia at 3 and 5 months after the therapy. Generally, with a median follow-up of 10 months, OS was 51.6%. Relapsed or residual B-ALL was identified in 3 patients who then received a second infusion of CAR-T-cells approximately 2–5·5 months after the first dose of CAR-T-cells. Unfortunately, they also did not respond to the second administration. The obtained results showed the promising potential of CAR-T-cells therapy in the treatment of relapsing/recurrent B-ALL CAR-T-cells therapy could be especially effective if CR and MRD-negative status are followed by allo-HSCT [86].

In 2017, Gardner et al. published the results of a phase I trial in which 43 children and young adults (between 1 and 27 years old) suffering from relapsing/remitting B-ALL were treated with a CAR-T-cell product, which had a defined composition of 1:1 CD4+/CD8+ CAR-T-cells (targeting CD19). MRD-negative CR was achieved in 41 (93%) patients. In 14 patients, the CAR-T administration was preceded by fludarabine and cyclophosphamide lymphodepletion. In these patients, the overall MRD-negative CR rate was 100%, while in the remaining patients who received cyclophosphamide-only lymphodepletion, it was 89%. Generally, the 12-month OS was 69.5%. In patients treated with fludarabine and cyclophosphamide lymphodepletion, the overall MRD-negative remission rate was 100%, while in the remaining patients, it was 89%. Overall, 45% of patients who achieved MRD-negative CR experienced relapse, with the CD-19-negative escape variant being accountable for 39% of relapses. Thus, it is expected that developing bispecific (against CD19 and CD22) CAR-T-cells could potentially reduce relapse rates and improve outcomes. At the dose of 1 × 10 × 6 CAR-T-cells per kg, dose-limiting toxicities occurred in 1 of the total 17 (9%) patients who received CAR-T therapy. It is worth noting that this patient underwent fludarabine/cytarabine lymphodepletion; thus, dose-limiting toxicities occurred in 1/6 (17%) of participants who received 2-agent lymphodepletion. CRS occurred in 40 of 43 (93%), and the rate of severe CRS (defined as the need for catecholamines, inotropes, or respiratory failure) was 23% (10 out of 43). However, the authors of the study emphasize that the adapted definition of CRS was stricter and when adapting criteria for severe CRS from other studies, grade 4 CRS has not been found in any patient. Therefore, this study demonstrated that CAR-T product with a fixed ratio of 1:1 of CD4+/CD8+ expressing cells is well tolerated and shows potential in treating relapsing/remitting B-ALL [87].

The effectiveness of tisagenlecleucel was tested during a phase II multicenter study by the Children’s Hospital of Philadelphia and the University of Pennsylvania. The study involved a group of 75 patients, aged 1 to 25 years, diagnosed with relapsed/refractory B-ALL, of which 46 underwent previous HSCT. Among 75 participants, 72 (96%) were given lymphodepleting chemotherapy (fludarabine and cyclophosphamide or cytarabine and etoposide). Generally, 55 of 75 experienced grade 3/4 adverse effects related to tisagenlecleucel, with CRS being the most frequent factor causing grade 3/4 toxicity in 35 (46.7%) patients. It is worth noting that the median time from CRS onset to the grade 3/4 levels was 3 days. Interestingly, after receiving the CAR-T infusion, tisagenlecleucel remained in the blood for up to 20 months. The MRD-negative CR was achieved in 81% of participants. OS and EFS were assessed at two time points after the administration of tisagenlecleucel. After 6 months from the infusion, OS and event-free survival (EFS) were 90% and 73%, respectively, while after one year, OS was 76% and EFS was 50%. The results of this first multicenter study of anti-CD19 CAR-T-cell-based therapies in relapsed/remitting B-ALL were consistent with previous single-center studies. Administration of tisagenlecleucel causes high rates of remission, which can be sustained in most of the cases. Further, CRS appears to be the most dangerous complication of the CAR-T treatment [85].

Ghorashian et al. generated a new variant of CAR-T-cells with a lower affinity binding for CD19 than a strong FMC63 binder, which was used in most of the previous studies [88]. Increased in vivo antitumor activity as well as in vitro cytotoxicity and proliferation of the new CAR-T-cells (CAT CAR-T-cells), as compared to the cells with FMC63 binder, was demonstrated. Furthermore, a clinical part of this study enrolled 14 patients with relapsing/remitting B-ALL. All patients underwent lymphodepletion consisting of fludarabine and cyclophosphamide CRS occurred in 13 patients (93%); however, there was no grade 3/4 CRS, which is a significant improvement in comparison to the other studies. Among patients who received CAT CAR-T infusions, 12 (86%) achieved molecular CR. In 11 of them, increased expansion and greater stability of CAR-T-cells were demonstrated compared to the available data on tisagenlecleucel [88,89]. The CR was maintained in 5 (37%) patients after 14 months; furthermore, in those patients circulating CAR-T-cells were detected. After 12 months, the EFS and OS were assessed, which were 46% and 65%, respectively. This is comparable to other studies in which tisagenlecleucel was used. However, the significant difference is that the patients in the trial by Ghorashian et al. did not proceed to HSCT, whereas in tisagenlecleucel studies, there was a significant group of patients who underwent HSCT after the treatment. The study showed that by mitigating the binding affinity of the CAR-T-cells, less toxicity of the treatment and a favorable safety profile can be achieved, as compared to the classic CAR-T-cells with stronger binders. Further, CAT CAR-T-cells are characterized by the increased expansion in patients, as they had blood mean maximal concentration 3× higher than tisagenlecleucel [88].

Due to the poor prognosis in relapses following the use of CD19 CAR-T-cells, a study was conducted to assess the effects of anti-CD22 CAR-T-cells. A total of 34 pediatric and adult patients, among whom 31 had failed previous anti-CD19 cell therapy, received an infusion from a relatively low dose of anti-CD22 CAR-T-cells. On the 30th day after the infusion, 24 (80%) of the 30 evaluated participants achieved CR or CR with incomplete count recovery, out of which 23 attained MRD-negativity. Four patients died during the treatment, and one of them developed grade 5 CRS. Nevertheless, in the remaining patients, the toxicity associated with CD22 CAR-T was low as no grade 3/4 events occurred. Among seven patients who did not receive any additional therapy, four had a relapse, as observed during the long-term follow-up. Nevertheless, 11 patients underwent HSCT, after which only 1 experienced relapse. The authors concluded that the anti-CD22 CAR-T lymphocytes are highly helpful in achieving a positive remission status in relapsed B-ALL patients, even in those who have previously received anti-CD19 CAR-T therapy [90].

Despite the efficiency of CAR-T treatment in B-ALL, applying this form of treatment in T-ALL patients still poses a significant challenge. Recently, in the results of a phase I study, 20 children and adults (aged 2–43 years) with relapsed/remitting T-ALL were given allogeneic donor-derived anti-CD7 CAR-T-cells. Importantly, patients who previously underwent HSCT (*n* = 12) were given CAR-T products from their donors (previous donor-derived—PDD patients), whereas participants who never underwent HSCT (*n* = 8) received CAR-T products from donors whose stem cells would be used for future HSCT (new donor-derived—NDD patients). The treatment was well tolerated as none of the participants experienced dose-limiting toxicities. Grade 3/4 CRS was observed in 2 patients. Overall, of the 20 patients, 19 responded to the treatment, and 18 participants achieved CR, out of which 17 were MRD-negative by day 15. The one patient who did not respond to the therapy (NDD group) lost CD7 expression. Other NDD patients proceed to the HSCT. The median follow-up time was 6.3 months, after which 15 patients remained in CR. The obtained results are promising and indicate that CAR-T treatment can be successfully implemented in T-ALL patients. The authors presented a novel strategy that relies on donor-derived CAR-T-cells. This approach was demonstrated to be highly effective, thus the need to further investigate the use of donor-derived CAR-T-cells in treatment not only in T-ALL but also in other hematological malignances [91]. Currently, a multicenter phase II study of anti-CD7 donor-derived CAR-T-cells for T-ALL is being conducted (NCT04689659).

CAR-T therapy can provide additional therapeutic options for patients with relapsing/remitting ALL. Improvement in CAR-T therapy may allow using this therapy as an independent therapeutic option without the need for additional forms of treatment such as HSCT. The results of the described studies in which CAR-T-cells therapy was used were briefly summarized in Table 2.

## 5. Other Therapy Options for Relapse/Chemoresistance ALL

The relapse rate of ALL patients is around 20%, and the remaining 10% of patients are still refractory to conventional therapy. Patients that have relapsed have an extremely poor prognosis; the 5-years OS is about 5–10% [92]. The prognosis of children with relapse ALL depends on the site of relapse, time from diagnosis to relapse, and genetic changes in blast cells [93]. Resistance to the initial glucocorticoid treatment is an important factor associated with significantly worse prognosis and increased risk of relapse. Steroid resistance has been linked with several mechanisms, such as mutations or polymorphisms in the human glucocorticoid receptor (*NR3C1*) gene, leading to disruption of glucocorticoid signaling. Disruption of proapoptotic/antiapoptotic signaling ratio in favor of the latter, related to alterations in B-cell lymphoma 2 (Bcl-2) protein family members expression, also contributes to the development of resistance to glucocorticoid treatment. Another cause of resistance to steroid treatment involves IKZF1 mutations in Ph-like ALL and signaling pathways overactivation, such as the interleukin-7 receptor (IL-7R) pathway and rat sarcoma virus (Ras) pathway in precursor B-cell ALL. Recently, the important role of the mitogen-activated protein kinase/extracellular signal-regulated kinase (MAPK-ERK) pathway was described, and its inhibitors were demonstrated to be effective in overcoming resistance to glucocorticoid treatment during the preclinical studies [94].

Purine metabolism significantly affects survival outcomes, as 6-Mercaptopurine is the basis of maintenance treatment. A number of gene mutations that lead to resistance to purines have been described. Examples include mutations in the PRPS1 gene, gain-of-function mutations in *NT5C2*, and loss of the *MSH6* gene. Various genetic aberrations such as chromosome translocations or gene rearrangements lead to the activation of additional metabolic pathways. In some of these cases, conventional chemotherapy can be combined with targeted therapy in order to improve the results of the treatment. However, the cancer cell is still able to develop a resistance mechanism that counters the action of those drugs [95]. Table 3 has shown ways of combining chemotherapy with different forms of treatment for relapsed/chemoresistant ALL.

Both children with relapse and a high risk of relapse are candidates for allogeneic HSCT [97]. Currently, these patients receive conventional chemotherapy to achieve remission. However, studies are currently underway to determine the superiority of blinatumomab or CAR-T-cell therapy over conventional chemotherapy.

With the advance of CART-cell therapy, studies began to investigate the efficacy of this therapy in patients with relapsed ALL compared to HSCT. In 2020, the results of a study on bone marrow transplantation after CART-cell therapy (post-HSCT) were published. The study by Zhang et al. included 120 patients with a median age of 12 years. The 1-year OS for patients with post-HSCT was 79.1%, and for patients without post-HSCT only 32.0% [98]. In another study conducted by Zhao et al., 122 patients ranging in age from 7 to 65 years were followed up. The 2-year OS for patients with post-HSCT was 77.0% vs. without post-HSCT 36.4% [99].

Currently, in oncological centers in Europe, a study by an Italian AIEOP group in collaboration with the German BFM group (NCT03643276) is underway to develop a new treatment protocol for children and adults with ALL. The study is being conducted in response to studies that have shown that chemotherapy today has become unacceptably toxic. This is especially true of patients who received intensified therapy due to the high risk of ALL recurrence. Its aim is the qualitative improvement of patients’ therapy, increasing OS, and the adjustment of risk stratification to the latest diagnostic markers with the use of available diagnostic methods. Expected changes to treatment protocols include the addition of blinatumomab as an alternative to conventional chemotherapy in patients with pre-B-ALL. In patients at high or intermediate risk of relapse, the use of intensive chemotherapy may lead to toxic effects. Therefore, AIEOP-BFM ALL-2017 aims to introduce blinatumomab as an adjunct to chemotherapy, especially for patients who respond poorly to its effects (NCT03643276) [31]. Polish hemato-oncology centers participate in this clinical trial.

## 6. Conclusions

Over the years, the treatment regimens of pediatric patients with ALL have undergone many changes. Despite the development of innovative techniques and the emergence of new pharmaceuticals, classic treatment methods such as chemotherapy or HSCT are still the basis of ALL treatment. Developing innovative methods of treatment and implementing them into protocols contribute to reducing the side effects of the therapy and OS. Nevertheless, targeted treatment can be applied only to a subset of patients. Therefore, the need for developing new treatments that will be effective in the widest possible group of ALL patients.

## Figures and Tables

**Table 1:** Comparison of selected treatment protocols in regard to prognostic factors, time points, and new approaches for treatment in clinical trials.

Protocol	Prognostic Factors	New Approaches for Treatment in Clinical Trials	Reference
AIEOP-BFM ALL 2017	Age at diagnosis < 1 year old Response to steroids on 8th Day of Protocol 1 FC-MRD in BM on 15th Day of Protocol 1 PCR-MRD in BM (TP1) on 33th Day of Protocol 1 PCR-MRD in BM (TP2) at 12 weeks of treatment genetic aberrations: *KMT2A-AFF1*, *TCF3-HLF*, *IKZF1*, *ETV6-RUNX1*, *TCF3-PBX1*, hypodiploidy	Patients with detectable resistance to chemotherapy and high risk of relapse may be candidates for treatment with blinatumomab (NCT03643276)	[31]
UK ALL 2011	WBC at diagnosis Age at diagnosis FC-MRD in BM on 8th,15th, and 29th Day of Protocol 1 PCR-MRD in BM at 9 and 14 weeks of treatment genetic aberrations: iAMP21, t(17;19) q(22,p13), KMT2A rearrangement, hypodiploidy	Patients with high risk may be candidates for chimeric antigen receptor T-cell therapy (CAR-T) as an alternative to HR blocks and HSCT (NCT03911128)	[32,33]
COG-AALL	WBC at diagnosis Age at diagnosis FC-MRD and PCR-MRD in BM on 15th and 29th Day of Protocol 1 genetic aberrations: *ETV6-RUNX1*, *KTM2A* gene rearrangements, t(4,11)	Patients with high risk may be candidates for a new drug called inotuzumab (AALL1732)	[34,35]
		Patients with high risk may be candidates for a blinatumomab (NCT03914625)	

AIEOP-BFM ALL 2017—International Collaborative Treatment Protocol for Children and Adolescents with Acute Lymphoblastic Leukemia, UK ALL 2011—United Kingdom National Randomized Trial For Children and Young Adults with Acute Lymphoblastic Leukemia and Lymphoma 2011, COG-AALL—Children Oncology Group Protocol; TP1—Time Point 1, TP2—Time Point 2, FC-MRD—flow cytometry minimal residual disease, PCR-MRD—polymerase chain reaction minimal residual disease, WBC—white blood cells, HR—high risk, BM—bone marrow.

**Table 2:** Results of various CAR-T protocols.

Therapy	Patients Characteristic	MRD-Negative CR Rate	EFS Rate	LFS Rate	OS Rate	Severe (Grade III/IV) CRS Rate	Ref.
Flu/Cy lymphodepletion + anti-CD19 CAR-T-cells	R/R B-ALL	60%	-	78.8% beginning at 4.8 months	51.6% 10-month OS	28.6%	[86]
Cy or Flu/Cy lymphodepletion + composition of 1:1 CD4+/CD8+ anti-CD19 CAR-T-cells	R/R B-ALL	− 93% in patients who received Cy alone − 100% in patients who received Flu/Cy	50.8% estimated 12-month EFS	-	69.5% estimated 12-month OS	23%	[87]
Flu/Cy or ara-C/ETP lymphodepletion + anti-CD19 CAR-T-cells	R/R B-ALL patients who previously underwent HSCT	81%	50% 12-month EFS	-	76% 12-month OS	46.7%	[85]
Flu/Cy lymphodepletion + anti-CD19 CAR-T-cells with a lower affinity binding	R/R B-ALL	86%	46% 12-month EFS	-	65% 12-month OS	0%	[88]
Flu/Cy lymphodepletion + anti-CD22 CAR-T-cells	R/R B-ALL patients, most of whom failed previous anti-CD19 CAR-T treatment	67.5%	-	58.1% 12-month LFS	-	3%	[90]
Flu/Cy lymphodepletion + anti-CD7 CAR-T-cells	R/R T-ALL	85%	-	83% after follow-up of 6.3 months	-	10%	[91]

MRD—minimal residual disease, EFS—event-free survival, LFS—leukemia-free survival, OS—overall survival, CRS—cytokine release syndrome, ref—reference, flu—fludarabine, cy—cyclophosphamide, CAR-T—chimeric antigen receptor redirected T-cells, R/R—relapsing/remitting, B-ALL—B-cell acute lymphoblastic leukemia, ara-c—cytarabine, ETP—etoposide, HSCT—hematopoietic stem cell transplantation, T-ALL—T-cell acute lymphoblastic leukemia.

**Table 3:** Different forms of combined therapy in ALL.

Chemotherapy Scheme	Brief Explanation	Examples
Sequential	Additional treatment is given after/prior to chemotherapy	Cranial radiotherapy [39,43] Blinatumomab therapy cycle after 2 blocks of consolidation chemotherapy [69] Blinatumomab (two cycles) after reinduction chemotherapy [69]
Concurrent	Additional treatment is given with conventional chemotherapy	Tyrosine kinase inhibitors (TKI) [52,53] Ruxolitinib [67] Rituximab [76] Epratuzumab [78]
Sandwiched	Additional treatment is given between chemotherapy cycles	Imatinib has been used in alternating schemes in some studies; however, concurring schemes were proved to be more effective [96]
Lymphodepletion	Chemotherapy applied prior to CAR-T-cells infusion. Increases effectivity and lowers toxicity of CAR-T treatment	Most of the CAR-T studies involve lymphodepletions prior to CAR-T-cells infusion [84,85,86,87,88,90,91]
